# Chondroitin Sulfate Alleviates Diabetic Osteoporosis and Repairs Bone Microstructure *via* Anti-Oxidation, Anti-Inflammation, and Regulating Bone Metabolism

**DOI:** 10.3389/fendo.2021.759843

**Published:** 2021-10-27

**Authors:** Shan Shan Qi, Meng Li Shao, Ze Sun, Si Min Chen, Ying Jun Hu, Xin Sheng Li, De Jing Chen, Hong Xing Zheng, Tian Li Yue

**Affiliations:** ^1^ College of Food Science and Engineering, Northwest Agriculture and Forestry (A&F) University, Yangling, China; ^2^ College of Biological Science and Engineering, Shaanxi University of Technology, Hanzhong, China; ^3^ Qinba State Key Laboratory of Biological Resources and Ecological Environment, Hanzhong, China; ^4^ QinLing-Bashan Mountains Bioresources Comprehensive Development C.I.C., Hanzhong, China; ^5^ Shaanxi Key Laboratory of Resource Biology, Hanzhong, China; ^6^ College of Food Science and Technology, Northwest University, Xi’an, China

**Keywords:** chondroitin sulfate, diabetes, osteoporosis, bone microstructure, bone metabolism

## Abstract

Diabetic osteoporosis (DOP) belongs to secondary osteoporosis caused by diabetes; it has the characteristics of high morbidity and high disability. In the present study, we constructed a type 1 diabetic rat model and administered chondroitin sulfate (200 mg/kg) for 10 weeks to observe the preventive effect of chondroitin sulfate on the bone loss of diabetic rats. The results showed that chondroitin sulfate can reduce blood glucose and relieve symptoms of diabetic rats; in addition, it can significantly increase the bone mineral density, improve bone microstructure, and reduce bone marrow adipocyte number in diabetic rats; after 10 weeks of chondroitin sulfate administration, the SOD activity level was upregulated, as well as CAT levels, indicating that chondroitin sulfate can alleviate oxidative stress in diabetic rats. Chondroitin sulfate was also found to reduce the level of serum inflammatory cytokines (TNF-α, IL-1, IL-6, and MCP-1) and alleviate the inflammation in diabetic rats; bone metabolism marker detection results showed that chondroitin sulfate can reduce bone turnover in diabetic rats (decreased RANKL, CTX-1, ALP, and TRACP 5b levels were observed after 10 weeks of chondroitin sulfate administration). At the same time, the bone OPG and RUNX 2 expression levels were higher after chondroitin sulfate treatment, the bone RANKL expression was lowered, and the OPG/RANKL ratio was upregulated. All of the above indicated that chondroitin sulfate could prevent STZ-induced DOP and repair bone microstructure; the main mechanism was through anti-oxidation, anti-inflammatory, and regulating bone metabolism. Chondroitin sulfate could be used to develop anti-DOP functional foods and diet interventions for diabetes.

## Introduction

There are 463 million people with diabetes worldwide currently, an average of 1 in 11 adults (20–79 years old), and by 2045, the diabetes population will jump to 700 million (1, 2). Long-term high blood glucose can cause microvascular injury, endangering the kidneys, heart, eyes, peripheral nerves, brain, feet, as well as bone (3).

Diabetic osteoporosis (DOP) is a bone complication caused by diabetes, characterized by lowered bone mineral density (BMD), changes of bone microstructure, and raised bone fragility. DOP greatly reduces the quality of living of patients, subjecting them to heavy economic burden (4). As the number of people with diabetes increases globally, the number of DOP is also increasing annually. Clinical data showed that about 50% to 65% of people with diabetes have decreased BMD and increased incidence of fractures, and nearly 35% of them have been diagnosed as osteoporosis (5).

The present approaches for DOP are oral hypoglycemic drugs or insulin injections, supplemented by calcium preparations, bisphosphonates, etc. (6). However, adverse drug reactions have always been a major challenge related to drug treatment goals. Therefore, exploring more safer and effective strategies are highly crucial.

Chondroitin sulfate (CS) is a natural sticky polysaccharide made from the cartilage of animals. The content of CS is different in the cartilage of different species and ages. As a drug for the treatment of joint diseases, it was used in conjunction with glucosamine to relieve pain and promote cartilage regeneration, which can fundamentally improve joint problems (7). It was reported that CS has anti-inflammation effects (8), has anti-psoriasis effects (9), enhances immunity (10), lowers blood lipid (11), and has anti-tumor effects (12). It also has a preventive effect on diabetic nephropathy.in streptozotocin (STZ)-induced diabetic mice (13), and CS was also reported to increase bone formation in ovariectomized rats (14). Whether it has a protective effect on DOP has not been studied. Our previous study found that the CS could increase the BMD of diabetic rats, but the mechanisms were not clear. So, in present study, we researched the protective effect and mechanisms of CS on DOP, which will provide a new approach for DOP treatment.

## Materials and Methods

### Chemicals

CS power was isolated and purified from the cartilage of giant salamander according to the method of Zhu (15); the content of CS in the experimental materials was 95%. STZ was obtained from Acmec Biochemical Company (Shanghai, China).

### Animals

Fifty-six-day old male rats were obtained from Cheng Du Dashuo Experimental Animal Company (Cheng Du, China, license no. SCXK 2020-030). Animals were kept in independent ventilated cages in a standardized animal room with constant temperature and humidity. Water and food intake of animals was not restricted. Animals were feed with diets prepared by the American Society of Nutrition (AIN93) standard. Animal experiment operations were approved by Shaanxi University of Technology Animal Ethics Committee (approval No. 2020-74).

### Diabetes Induction in SD Rats, Animal Grouping, and Treatment

After 7 days of acclimatization, except for 10 animals in the control group, the remaining animals were intraperitoneally injected with 45 mg/kg of STZ after fasting overnight, and the dosage of STZ was based on our previous study (16, 17); STZ was dissolved in citric acid buffer solution at pH 4.3, and 10 animals in the control group were injected with citric acid buffer solution. After 72 h, animal’s blood glucose was detected, and animals with blood glucose higher than 11.1 mmol/L were selected for subsequent experiments.

Then, animals were regrouped into four groups: Group 1, control group (*n* = 10), rats were given deionized water by gavage every day; Group 2, type 1 diabetic group (*n* = 10), named T1DM group, type 1 diabetic rats were given deionized water by gavage every day; Group 3, CS-treated group (*n* = 10), named CS group, type 1 diabetic rats were given CS (200 mg/kg/day) by gavage every day; Group 4, metformin group (*n* = 10), named Met group, type 1 diabetic rats were given metformin (200 mg/kg/day) by gavage every day. The dose of CS was selected by pre-experiment.

During the experiment, animals were weighed every week, water and diet of animals were recorded, and the blood glucose of animals was measured every week. After 10 weeks of administration, animals were anesthetized with isoflurane and were sacrificed by cervical dislocation; blood was collected, and serum was separated and stored at −80°C. At the same time, femurs, vertebrae, and tibias of animals were collected.

### BMD Measurement

The BMD of femur and vertebrae of each rat were obtained by using small-animal dual-energy X-ray absorptiometry (InAlzyer, Korea).

### Bone Micro-CT Measurement

Femur tissues of rats were collected and scanned by a Locus SP micro-CT (GE Healthcare, Danderyd, Sweden) with a resolution of 6.5μm. The processing and analysis software were MICVIEW 3D reconstruction processing software and ABA-specific bone analysis software. The data of cortex volume, bone surface area, trabecular number, and bone volume were obtained by using ABA-specific bone analysis software.

### Bone Turnover Marker Detection

According to instructions listed in ELISA test kits (Elabscience, Wuhan, China), a microplate reader (Elx808, Winooski, USA) was used to detect the content of bone turnover markers (CTX-1, OPG, ALP, TRACP 5b, RANKL, osteocalcin, and RUNX 2) in serum of each group of rats.

### Oxidative Stress Index Detection

According to instructions listed in kits (Beyotime, Shanghai, China), SOD activity, MDA content, CAT activity, and GSH content in serum were detected by an ultraviolet and visible spectrophotometer (Alpha1860S, Shanghai, China).

### Inflammatory Cytokine Detection

Based on instructions listed in ELISA test kits (Elabscience, Wuhan, China), the microplate reader (Elx808, Winooski, USA) was used to detect the content of serum inflammatory cytokines (IL-6, TNF-α, MCP-1, and IL-1).

### Pathological Analysis of Femur Bone Tissue

Femur of each rat was fixed in 3.8% paraformaldehyde solution for 48 h, then rinsed with PBS, placed in 10% EDTA solution for 5 weeks, and then made into 4-micron-thick paraffin sections by a tissue slicer (Leica, Wetzlar, Germany), and the sections were stained with hematoxylin and eosin, sealed with neutral gum. After that, a slide was placed under a microscope to observe femur pathological changes. Bone morphometric parameters including trabecular separation (Tb·Sp), bone volume per tissue volume (BV/TV), and trabecular thickness (Tb·Th) were analyzed by image pro plus (IPP) 6.0 software.

### Pathological Observation of Tibia Bone Marrow Adipocytes

Tibia of each rat was fixed in 3.8% paraformaldehyde solution for 48 h, then rinsed with PBS; femur was placed in 10% EDTA solution for 5 weeks and then made into 4-micron-thick paraffin sections by a tissue slicer (Leica, Wetzlar, Germany), and sections were stained with hematoxylin and eosin, sealed with neutral gum. After that, the slide was placed under a microscope to observe bone marrow adipocytes in tibia. Adipocytes were counted in each field, and the diameter of adipocytes was measured with IPP 6.0 software.

### Femur Osteoclast Observation-TRAP Staining

Four-micron-thick femur paraffin sections were stained with TRAP staining kit, based on the instructions listed in the kit. The stained slides were sealed with neutral gum. After that, a slide was placed under a microscope (200× magnification) to observe osteoclasts in the femur. Osteoclasts were purple-red after staining, five fields of view were selected for each slice, and the number of osteoclasts in each field was counted.

### Immunohistochemical

Femur paraffin slides (4 μm thick) were soaked in xylene for 20 min, and then slides were dehydrated with gradient ethanol and placed in a water bath at 95°C in citrate antigen retrieval solution (pH 6) for 1 h. After cooling to room temperature, slides were washed with PBS; 1% BSA solution was added to the slides to block endogenous peroxidase, and then the slides were washed with PBS, incubated with primary antibodies (OPG, RANKL, and RUNX2, respectively) (Santa Cruz Biotech, USA) for 1.5 h at 37°C in a constant temperature incubator, double washed with PBS, incubated with secondary antibody (Santa Cruz Biotech, USA) for 2 h, washed with PBS three times, and then incubated with DAB. After that, slides were counterstained with hematoxylin, and sealed with neutral gum. IHC-stained slides were observed under a microscope (Olympus, Germany) with 200× magnification. Five fields of view were chosen for each slice, and IPP software was used to count the area of positive staining, and percentage of positive area was calculated.

### Statistical Analysis

Statistical analyses were performed with ANOVA by SPASS 19.0; all of the data were shown as mean ± SD, and statistical significance was compared between groups using the LSD method. *p* < 0.05 was considered significant, and *p* < 0.01 was considered extremely significant.

## Results

### Chondroitin Sulfate Relieved the Symptoms of Hyperglycemia, Polydipsia, and Polyphagia Caused by Diabetes in SD Rats

**Figure 1:**
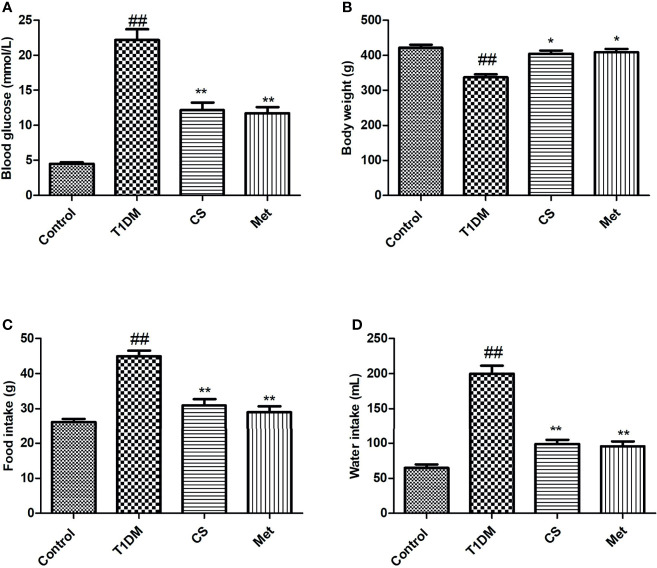
Blood glucose, body weight, food intake, and water intake at the end of the 10th week in different groups. **(A)** Blood glucose in different groups. **(B)** Body weight in different groups. **(C)** Food intake in different groups. **(D)** Water intake in different groups. ^##^indicates *p* < 0.01 compared with control; **indicates *p* < 0.01 compared with the diabetic group; *indicates *p* < 0.05 compared with the diabetic group.

The blood glucose, water intake, and food intake in type 1 diabetic rats were significantly increased compared with control (*p* < 0.01) (**Figures 1A, C, D**), while the body weight was decreased (**Figure 1B**); this result was consistent with basic pathological changes of diabetes, indicating that the type 1 diabetes animal model was successfully established. After 10 weeks of CS or metformin administration, the symptoms of diabetes in rats were effectively alleviated, reflected in lowered blood glucose, increased body weight, and reduced water and food intake. This indicates that CS can reduce blood glucose of diabetic rats and relieve the symptoms of diabetes.

### Chondroitin Sulfate Increased BMD of Type 1 Diabetic Rats

**Figure 2:**
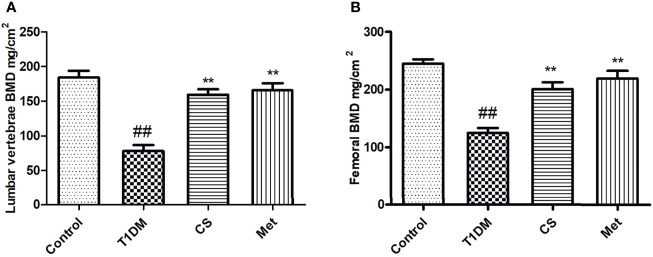
The bone mineral density of different groups at the end of the 10th week. **(A)** The BMD of lumbar vertebrae in different groups. **(B)** The femoral BMD in different groups. ^##^indicates *p* < 0.01 compared with control. **indicates *p* < 0.01 compared with the diabetic group.

Compared with control group, the BMD (lumbar vertebrae and femur) in diabetic rats was lowered significantly (*p* < 0.01) (**Figure 2**). After 10 weeks of CS or metformin administration, the BMD in lumbar vertebrae and femur was increased (CS *vs*. T1DM group, *p* < 0.01; Metformin *vs*. T1DM groups, *p* < 0.01) (**Figure 2**), indicating that CS could increase the BMD of type 1 diabetic rats.

### Chondroitin Sulfate Repaired Bone Micro-CT Structure of Type 1 Diabetic Rats

**Figure 3:**
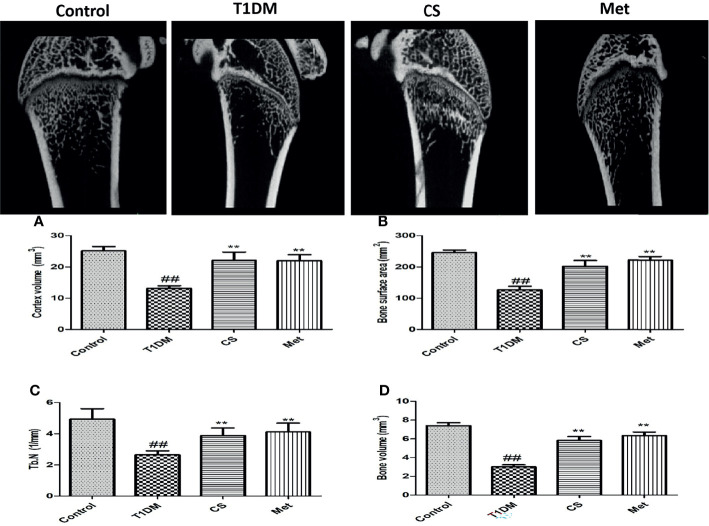
**(A)** The femur micro-CT structure of different groups at the end of the 10th week. **(B)** Cortex volume in different groups. **(C)** Bone surface area in different groups. **(D)** Trabecular number in different groups. **(E)** Bone volume in different groups. ^##^indicates *p* < 0.01 compared with control. **indicates *p* < 0.01 compared with the diabetic group.

Femur micro-CT scanning results showed that trabecular structure in the model group was sparse, and some trabecular areas disappeared (**Figure 3A**). After 10 weeks of administration of CS or metformin, trabecular structure was repaired. Micro-CT metrological data (**Figures 3A–E**) showed that cortical bone volume, bone surface area, number of bone trabecular, and bone volume in type 1 diabetic group were significantly lower than those in the control group (*p* < 0.01). After 10 weeks of CS or metformin administration, the above indexes were upregulated, and the differences were significant compared with the diabetic group.

### Chondroitin Sulfate Regulated Bone Turnover of Type 1 Diabetic Rats

As **Table 1** shows, some of the serum bone turnover markers (OPG, RUNX 2, osteocalcin, and TRACP 5b) and OPG/RANKL ratio levels were lowered in type 1 diabetic rats compared with control (*p* < 0.01), and those were significantly increased in the CS and Met group compared with the type 1 diabetic group. Other serum bone turnover markers such as RANKL, CTX 1, and ALP levels were higher in the type 1 diabetic group compared with the control (*p* < 0.01), which were significantly decreased in CS and Met groups, indicating that CS could regulate bone turnover of type 1 diabetic rats.

**Table 1:** Bone turnover markers in different groups at the end of the 10th week.

	Control	T1DM	CS	Met
OPG (ng ml^−1^)	9.31 ± 2.07	2.88 ± 1.05^##^	7.16 ± 2.25^**^	7.56 ± 2.72^**^
RANKL (ng ml^−1^)	3.08 ± 1.12	10.20 ± 2.50^##^	4.36 ± 1.31^**^	5.80 ± 2.07^**^
OPG/RANKL ratio	3.02 ± 1.23	0.29 ± 0.11^##^	2.14 ± 0.96^**^	3.02 ± 1.23^**^
RUNX 2 (ng ml^−1^)	10.69 ± 2.96	3.64 ± 1.36^##^	8.49 ± 2.71^**^	8.42 ± 2.73^**^
Osteocalcin (ng ml^−1^)	30.11 ± 6.67	9.60 ± 2.95^##^	23.31 ± 5.73^**^	24.24 ± 4.75^**^
TRACP 5b (U dl^−1^)	3.25 ± 1.31	6.47 ± 1.50^##^	4.92 ± 1.11^*^	4.59 ± 1.24^*^
ALP (U dl^−1^)	75.93 ± 13.15	167.31 ± 18.55^##^	96.99 ± 19.86^**^	92.90 ± 14.31^**^
CTX 1 (ng ml^−1^)	41.73 ± 9.68	107.14 ± 23.21^##^	60.18 ± 16.38^**^	54.39 ± 17.35^**^

^##^indicates p < 0.01 compared with control, **indicates p < 0.01 compared with the diabetic group, *indicates p < 0.05 compared with the diabetic group.

### Chondroitin Sulfate Downregulated Inflammatory Cytokines of Type 1 Diabetic Rats

**Figure 4:**
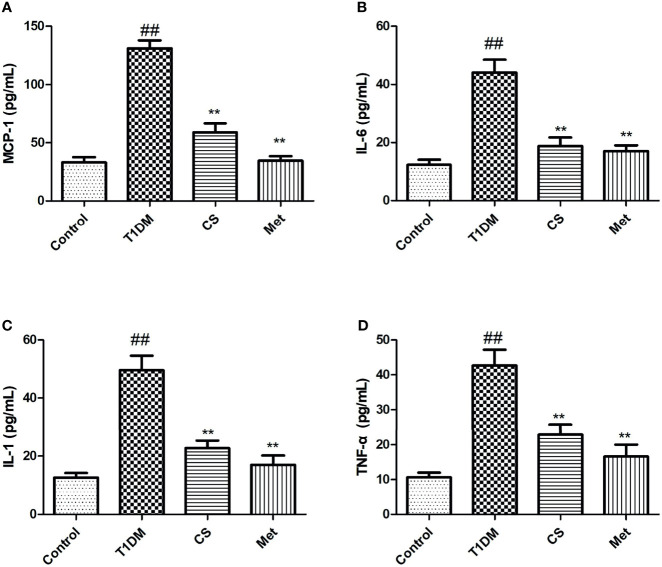
The serum inflammatory cytokine levels in different groups at the end of the 10th week. **(A)** MCP-1 levels in serum; **(B)** IL-6 levels in serum; **(C)** IL-1 levels in serum; **(D)** TNF-a levels in serum. ^##^indicates *p* < 0.01 compared with control. **indicates *p* < 0.01 compared with the diabetic group.

Serum inflammatory cytokine levels in type 1 diabetic rats increased dramatically compared with the control group (*p* < 0.01). After 10 weeks of CS or metformin treatment, the serum inflammatory cytokine levels were lowered in CS or Met groups (CS *vs*. T1DM group, *p* < 0.01; Metformin *vs*. T1DM groups, *p* < 0.01) (**Figure 4**), indicating that CS has an inhibitory effect on inflammation induced by type 1 diabetes in SD rats.

### Chondroitin Sulfate Alleviated Oxidative Stress in Type 1 Diabetic Rats

**Figure 5:**
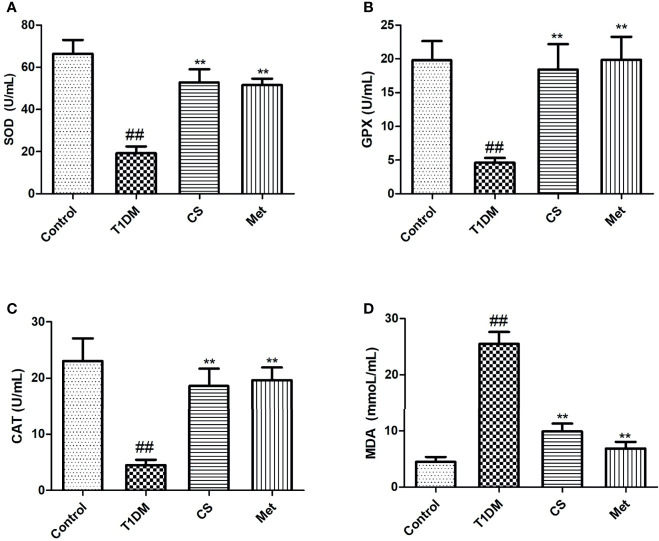
The level of serum oxidative stress parameters in different groups at the end of the 10th week. **(A)** The level of SOD activities in different groups. **(B)** The level of glutathione peroxidase (GPX) activities in different groups. **(C)** The level of catalase (CAT) activities in different groups. **(D)** The level of malondialdehyde (MDA) content in different groups; ^##^indicates *p* < 0.01 compared with control, **indicates *p* < 0.01 compared with the diabetic group.

Oxidative stress existed in diabetic rats; serum SOD, GPX, and CAT activity levels were lowered, and MDA level was raised in type 1 diabetic rats. Ten weeks after CS or metformin treatment, SOD, GPX, and CAT activity levels were raised, and MDA level was lowered (CS *vs*. T1DM, *p* < 0.01; Met *vs*. T1DM, *p* < 0.01) (**Figure 5**). The results indicated that CS could alleviate oxidative stress in T1DM rats.

### Chondroitin Sulfate Repaired Bone Microstructure of Type 1 Diabetic Rats

**Figure 6:**
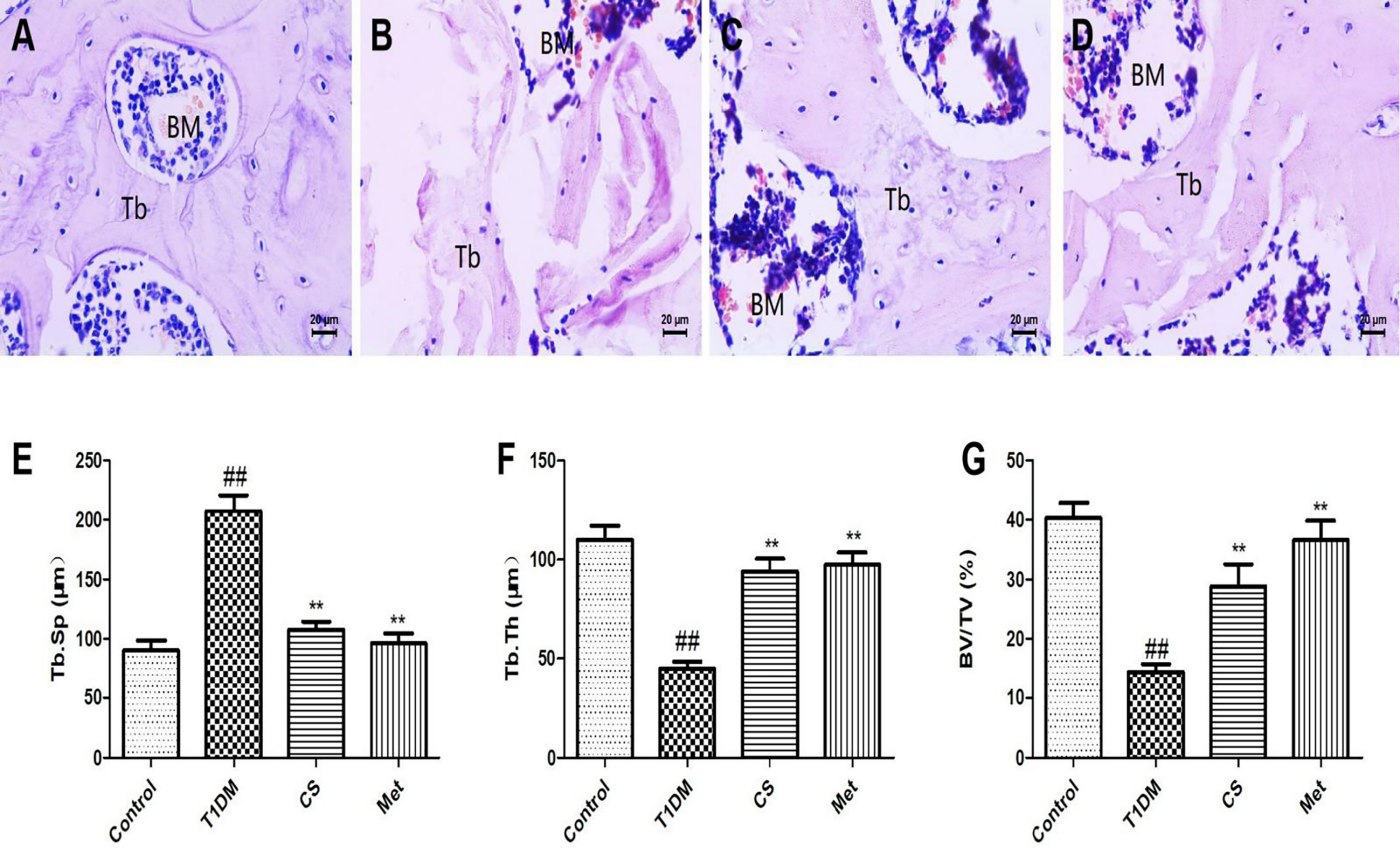
The pathological sections of femur of rats in each group at the end of the 10th week; paraffin section of femur was stained with hematoxylin and eosin, 400×. **(A)** The control group femur. **(B)** The diabetic group femur. **(C)** The CS group femur. **(D)** The Met group femur. **(E)** The trabecular separation (Tb·Sp) in different groups. **(F)** The femoral thickness (Tb·Th) in different groups. **(G)** The percentage of bone trabecular area (BV/TV) in different groups; ^##^indicates *p* < 0.01 compared with control. **indicates *p* < 0.01 compared with the diabetic group. BM. Bone marrow; Tb. Trabecular bone.

Compared with the control group, the femoral bone of diabetic rats was sparse and fractured, the spacing of trabecular bone became wider, and the trabecular bone became thinner (**Figures 6A–D**). The femoral structure was repaired after 10 weeks of CS or metformin administration. The results of bone morphometric data (**Figures 6E–G**) showed that the femoral thickness (Tb·Th) and the percentage of bone trabecular area (BV/TV) in CS or metformin treatment groups were significantly increased compared with the type 1 diabetic group (*p* < 0.01), and the trabecular separation (Tb·Sp) was decreased in CS or metformin treatment groups compared with the type 1 diabetic group (*p* < 0.01).

### Chondroitin Sulfate Inhibited Bone Marrow Lipogenesis of Type 1 Diabetic Rats

**Figure 7:**
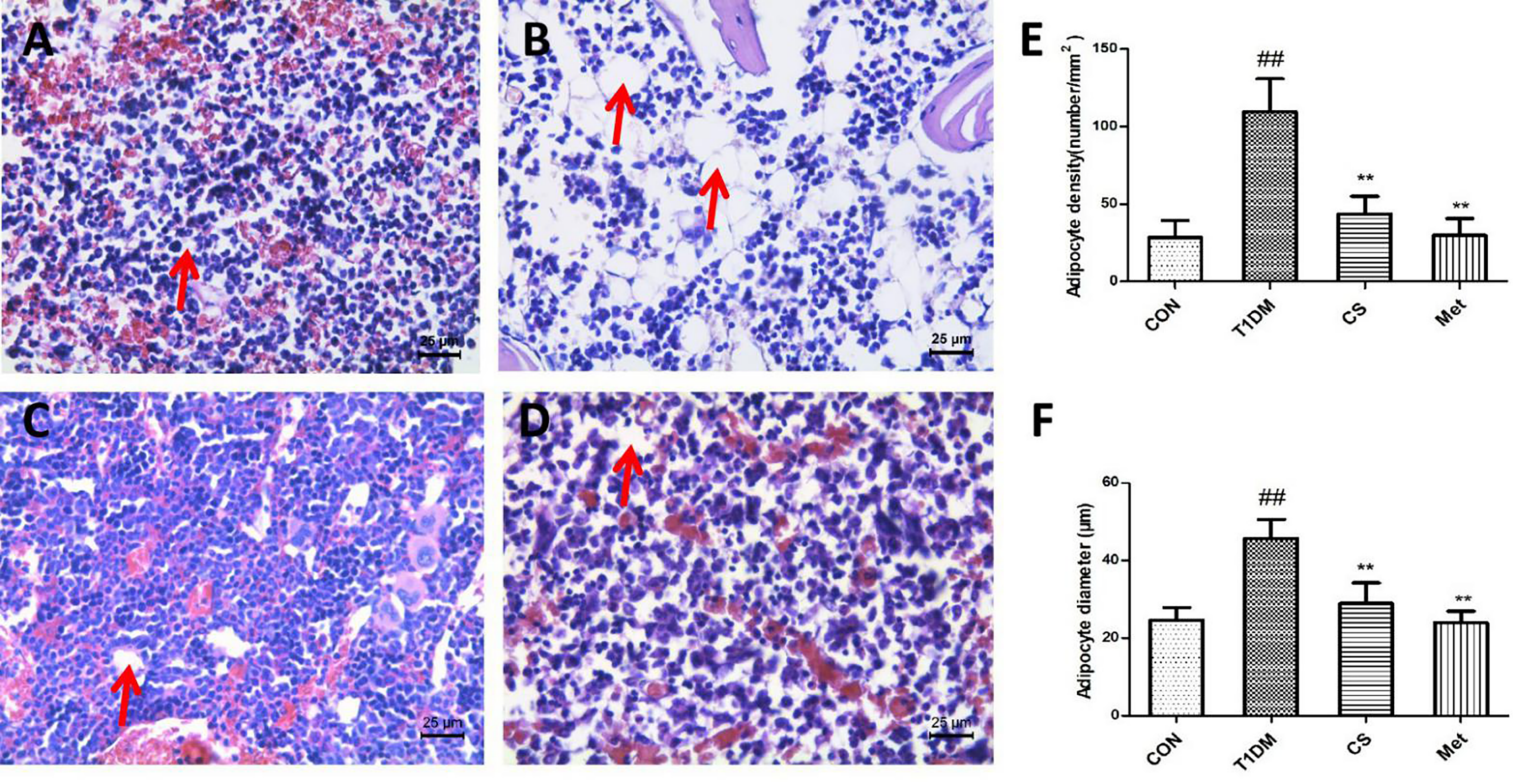
Adipocytes in the bone marrow cavity of the tibia in different groups at the end of the 10th week, H&E staining. **(A)** The control group tibia bone marrow. **(B)** The diabetic group tibia bone marrow. **(C)** The CS group tibia bone marrow. **(D)** The Met group tibia bone marrow. **(E)** Adipocyte density in different groups. **(F)** Adipocyte diameter in different groups. ^##^indicates *p* < 0.01 compared with control. **indicates *p* < 0.01 compared with the diabetic group. Red arrows indicate adipocytes.

Compared with the control group, the number of bone marrow adipocytes of diabetic rats increased significantly, and the bone marrow adipocyte density and adipocyte diameter were larger than the control (*p* < 0.01) (**Figure 7**). Ten weeks after CS or metformin treatment, the bone marrow adipocyte density and adipocyte diameter decreased (CS *vs*. T1DM, *p* < 0.01; Met *vs*. T1DM, *p* < 0.01) (**Figure 7**). The results indicated that CS could inhibit bone marrow lipogenesis of type 1 diabetic rats.

### Chondroitin Sulfate Inhibited Osteoclastogenesis of Type 1 Diabetic Rats

**Figure 8:**
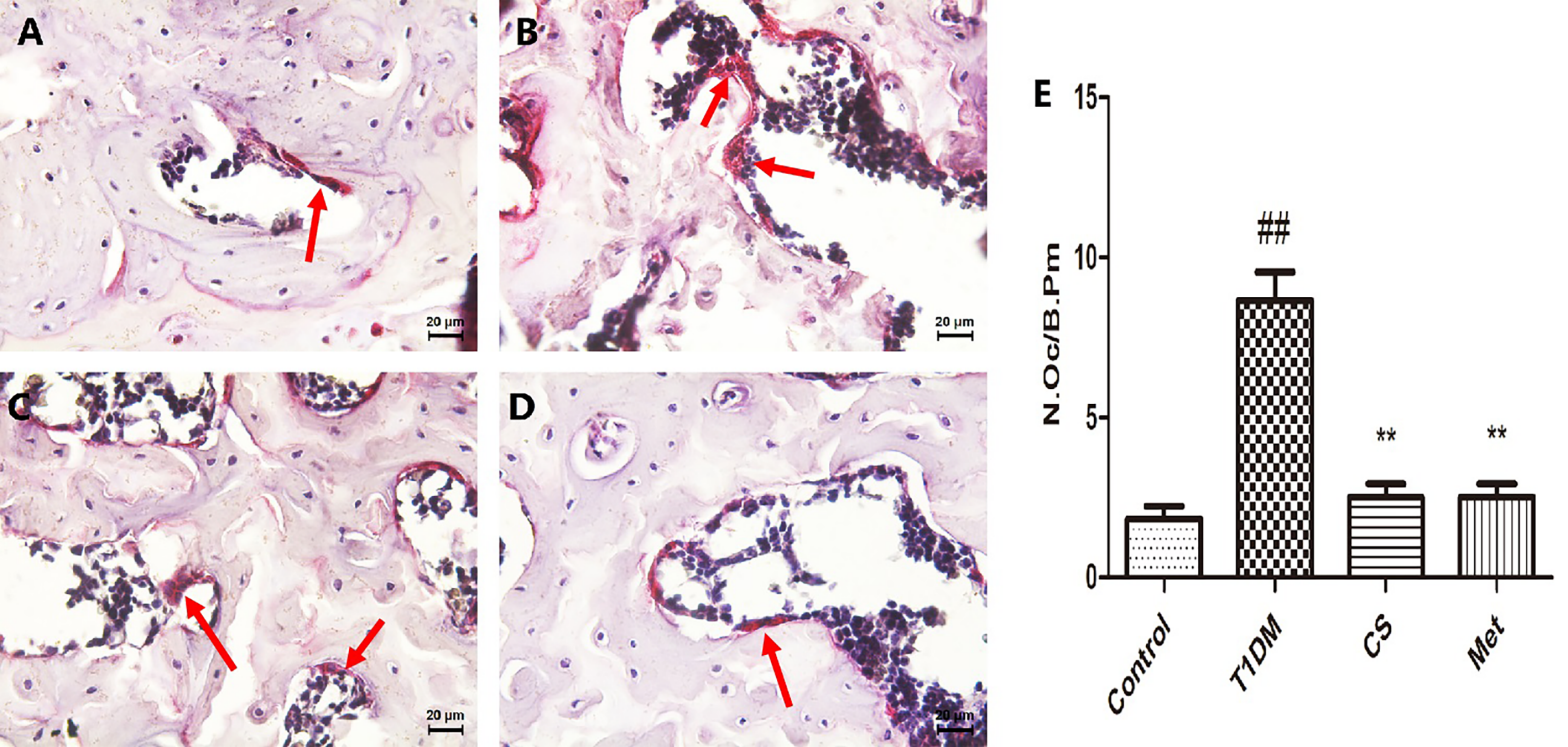
The osteoclasts in femoral bone tissues of different groups at the end day of the 10th week. **(A)** The femoral bone tissues of control group rat. **(B)** The femoral bone tissues of diabetic group rat. **(C)** The femoral bone tissues of CS group rat. **(D)** The femoral bone tissues of Met group rat. **(E)** Number of osteoclasts per unit field in different groups. Bone tissues were stained by TRACP method, osteoclasts are multinucleate cells, and the red arrows point to osteoclasts. ^##^indicates *p* < 0.01 compared with control. **indicates *p* < 0.01 compared with the diabetic group.

After TRAP staining, the osteoclasts were stained purplish red. There was an increased number of femoral osteoclasts in type 1 diabetic group rats compared with control (*p* < 0.01) (**Figure 8**). Ten weeks after CS or metformin treatment, osteoclast number was decreased in the CS or Met group (CS group *vs*. T1DM group, *p* < 0.01; Met group *vs*. T1DM group, *p* < 0.01) (**Figure 8E**), indicating that CS could inhibit osteoclasts proliferation in type 1 diabetic rats.

### Chondroitin Sulfate Upregulated OPG and RUNX 2 and Downregulated RANKL Expression in Bone Tissues

**Figure 9:**
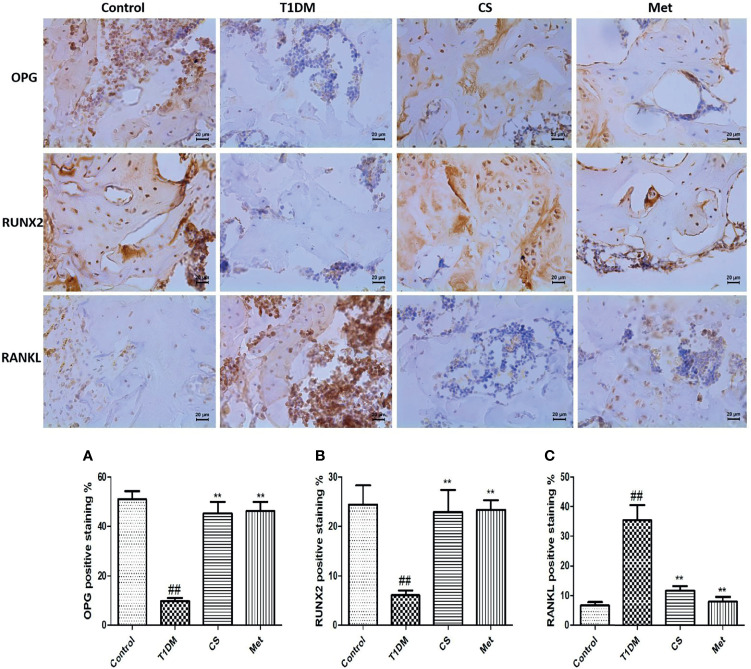
Immunohistochemical staining of femur and positive staining percentage of OPG, RUNX2, and RANKL in each group. **(A)** The positive staining percentage of OPG in the femur of each group. **(B)** The positive staining percentage of RUNX 2 in the femur of each group. **(C)** The positive staining percentage of RANKL in the femur of each group. ^##^indicates *p* < 0.01 compared with control. **indicates *p* < 0.01 compared with the diabetic group.

Pathological analysis showed that bone OPG and RUNX 2 expression levels were lowered in type 1 diabetic rats (T1DM *vs*. Control, *p* < 0.01); RANKL level in bone tissue was higher in type 1 diabetic rats (T1DM *vs*. Control, *p* < 0.01) (**Figure 9**). After 10 weeks of CS or metformin administration, the bone OPG and RUNX 2 levels increased (CS or Met group *vs*. T1DM group, *p* < 0.01) and the RANKL level decreased (CS or Met *vs*. T1DM group, *p* < 0.01) (**Figures 9A–C**).

## Discussion

DOP is a serious metabolic bone disease associated with diabetes. It has the characteristics of reduced BMD, destruction of bone microstructure, increased brittleness, reduced strength, and easy to fracture, which seriously affects the quality of life of people with diabetes (18, 19). Clinical treatment of DOP is to use chemical drugs such as vitamin D3, diphosphonate, calcitonin, and calcium to inhibit bone absorption, promote bone formation, and improve bone mineralization on the premise of controlling blood glucose. However, using chemical drugs long term can also lead to side effects, such as gastrointestinal diseases (20). Therefore, natural anti-osteoporosis drugs are much popular.

In the present study, we used the method of STZ injection to construct a rat model of type 1 diabetes, and intragastric administration of CS for 10 weeks. We detected the BMD, bone micro-CT, and bone pathology, and the results indicated that CS could inhibit bone loss, increase BMD, repair bone microstructure of type 1 diabetic rats, and thus prevent DOP.

DOP is closely related to oxidative stress. Hyperglycemia induced reactive oxygen species (ROS) production *in vivo* and hindered the proliferation and differentiation of osteoblasts (21–23). It has been confirmed that a sharp increase in the level of ROS induces death of osteoblasts, resulting in bone structure damage and BMD reduction (24). In the present study, we found that the SOD, GPX, and CAT activity levels were upregulated after 10 weeks of CS administration, indicating that CS could inhibit oxidative stress in type 1 diabetic rats; this may be one of important reasons why CS could prevent bone loss in type 1 diabetic rats.

Inflammation is closely related to osteoporosis, which is another main cause of DOP (25). The level of inflammatory cytokines in diabetes was significantly higher than that in healthy people, and accumulation of inflammatory cytokines could mediate oxidative stress damage, prompting osteoclast proliferation, increasing bone absorption, and thus causing osteoporosis (26). In this study, we found that CS could inhibit the inflammation in type 1 diabetic rats, and inflammatory cytokines were downregulated by CS. Many other natural products have also been reported to relieve DOP through an anti-inflammatory manner (23, 27–29).

Both osteoblasts and bone marrow adipocytes were derived from bone marrow mesenchymal stem cells (30). As the number of bone marrow adipocytes increased, that of osteoblasts will decrease. Changes in the number and size of bone marrow adipocytes were positively correlated with osteoporosis, so they were important criteria for evaluating the efficacy of osteoporosis drugs (31–33). In diabetes, hyperglycemia promotes the differentiation of bone marrow adipocytes and inhibits osteoblast differentiation (34). As indicated in the present study, the bone marrow adipocyte number and size in type 1 diabetic rats were higher than those in control group rats, which were downregulated by 10 weeks of CS administration. Our previous study also found that trace elements of zinc, black rice anthocyanin, and lycopene were all having a preventive effect on DOP, and all of them can inhibit bone marrow adipocyte generation (4, 23, 35), which is consistent with the results of this study.

Bone tissue structure is an important basis for evaluating bone health (36). In this study, we applied bone micro-CT and bone tissue pathology techniques to evaluate bone structure; the results indicated that CS can improve bone structure lesions caused by diabetes, and it can effectively restore bone morphological parameters and upregulate BMD.

The biochemical markers of bone turnover (BMBT) were metabolite of bone tissue. BMBT includes bone formation and bone resorption markers, which were important indicators for laboratory diagnosis of osteoporosis (37, 38). Not only can it quickly reflect the process of bone formation and bone resorption, but it also can be used to reveal pathogenesis of metabolic bone disease, predict the rate of bone loss and fracture risk, and diagnose osteoporosis; at the same time, it can be used to quickly reflect the therapeutic effect of anti-osteoporosis drugs (39). In our present study, the bone formation markers (osteocalcin and ALP) as well as bone resorption markers (TRACP 5b and CTX 1) all increased in type 1 diabetic rats, which indicated that diabetic rats had a higher bone turnover rate. This study showed that CS could downregulate the BMBT in diabetic rats and inhibit the higher bone turnover rate.

RANKL was a member of the TNF superfamily involved in immune regulation and bone metabolism, and it was an important activator of osteoclast differentiation and maturation (40). RANKL activates osteoclast differentiation, thus promoting bone resorption by competing with OPG to bind to receptor RANK. Overexpression of RANKL can lead to excessive activation of osteoclasts, which can lead to osteoporosis (41). OPG was also a member of the TNF superfamily. It was highly expressed in testis and bone marrow (42). OPG binds to RANKL and blocks binding of RANKL to RANK. At the same time, OPG can inhibit osteoclast differentiation and maturation, and promote osteoclasts apoptosis (43). When the ratio of OPG to RANKL increases, bone formation activity of osteoblasts increases, and bone metabolism tends to be in a positive balance. When the ratio of OPG to RANKL decreases, the bone resorption activity of osteoclasts increases, and bone metabolism tends to be in a negative balance (44). In the present study, the level of OPG/RANKL ratio in diabetic rats was greatly decreased compared with control, indicating increased bone resorption activity; after 10 weeks of CS treatment, the OPG/RANKL ratio was upregulated, indicating increased bone formation activity. One of the mechanisms of CS against DOP is by upregulating the OPG/RANKL ratio. In this study, we detected the bone OPG, RANKL, and RUNX 2 proteins using an immunohistochemical method, which has the advantage of localizing the protein, but in a quantitative aspect, it is inferior to Western blotting; in subsequent experiments, we will supplement the Western blotting experiment to confirm the above results.

As a first-line drug for diabetes, metformin is a chemically synthesized drug. The main side effects are gastrointestinal reactions, such as nausea and vomiting (45), as well as gut microbiota dysbiosis (46). Secondly, metformin will interfere with the absorption of vitamin B12 and folic acid. Studies have shown that up to 30% of patients taking metformin have B12 deficiency (47, 48). In this study, CS showed a good anti-DOP effect, similar to metformin. However, the CS used in this study was naturally extracted and has the advantages of high safety and less side effects compared with metformin. This study revealed the role of CS in DOP treatment through animal experiments. Later, we will further reveal its molecular mechanism through *in vitro* cell experiments and further confirm its effect through human experiments.

## Data Availability Statement

The original contributions presented in the study are included in the article/Supplementary Material. Further inquiries can be directed to the corresponding authors.

## Ethics Statement

The animal study was reviewed and approved by Shaanxi University of Technology Animal Ethics Committee.

## Author Contributions

Study design: SQ, HZ, DC, TY, and XL. Study conduct: SQ, HZ, MS, ZS, YH, and SC. Data collection and analysis: SQ, HZ, MS, ZS, YH, and SC. Animal model construction: SQ and HZ. Histopathological experiment: SQ, MS, ZS, and SC. Manuscript draft: SQ. Manuscript revision: HZ. All authors contributed to the article and approved the submitted version.

## Funding

This research was funded by the Postdoctoral Program in Shaanxi University of Technology (SLGBH16-03), “Shaanxi Sanqin Scholar” Innovation Team, the Key Research Projects of Shaanxi Provincial Department of Education (20JY005), Shaanxi University of Technology Project (SLGKY2010), and the Key projects of Shaanxi Science and Technology Department (2021FP-14), and Shaanxi University of Technology Project (SLGKY2004).

## Conflict of Interest

The authors declare that the research was conducted in the absence of any commercial or financial relationships that could be construed as a potential conflict of interest.

## Publisher’s Note

All claims expressed in this article are solely those of the authors and do not necessarily represent those of their affiliated organizations, or those of the publisher, the editors and the reviewers. Any product that may be evaluated in this article, or claim that may be made by its manufacturer, is not guaranteed or endorsed by the publisher.
